# The Rational Contrast Between Del Nido Solution and the Approaches on Minimally Invasive Extracorporeal Technologies

**DOI:** 10.21470/1678-9741-2023-0194

**Published:** 2024-01-30

**Authors:** Ignazio Condello

**Affiliations:** 1 Department of Cardiac Surgery, Anthea Hospital, GVM Care & Research, Bari, Italy. E-mail: ignicondello@hotmail.it

Time has fascinated the history of humanity since its origins: in particular, preand post-Aristotelian philosophy, Galilean science, Albert Einstein’s theory of relativity, and Enrico Fermi’s quantum physics. The space-time in the theory of relativity is an indissoluble variable - there is no space without time and vice versa. Organ protection from ischemia is a matter of time in relation to the technique; there are many different cardioplegia solutions of varying compositions used for myocardial arrest and protection in cardiac surgery, and the choice of cardioplegia is often up to institution or surgeon preference. Most conventional blood cardioplegia (BC) requires a dose every 15 to 20 minutes; del Nido (DN) cardioplegia solution was originally developed for pediatric and congenital heart surgery and was widely adopted for its ability to provide myocardial protection for 90 minutes after a single induction dose. The solution uses lidocaine and magnesium to arrest the myocardium in a depolarized state. Haci Ali Ucak et al.^[1]^ compared DN solution with BC in aortic valve replacement in a two-year single-institute retrospective cohort study. Subjects who underwent aortic valve replacement surgery were divided into two groups (DN and BC), and outcomes were compared. The results of cardiopulmonary bypass (CPB) time were statistically significantly shorter in the DN group (BC 60.8 ± 18.5 min., DN 53.7 ± 15.2 min.) (*P*=0.046). The rate of postoperative use of intravenous positive inotropic support drugs (dopamine, dobutamine, norepinephrine, etc.) for more than two hours was significantly higher in the BC group (20 [23.5%] in the BC group and nine [17.3%] in the DN group) (*P*=0.035). In the context of minimally invasive extracorporeal circulation (MiECC), we share our opinion on DN solution during aortic valve replacement procedures in the adult cardiac surgery. MiECC is a specified technology that integrates all contemporary advancements in perfusion science by comprising certain components: a closed circuit with biologically inert blood contact surfaces and reduced priming volume; a centrifugal pump; a membrane oxygenator; a heat exchanger; a venous bubble trap or venous air removing device; a cardioplegia system; and a shed blood management device^[2]^.

In our experience the use of DN solution increases temporarily hemodilution in extracorporeal circulation and is a variable that acts directly on colloid oncotic pressure (COP), determined by all plasma proteins in the intraand extravascular compartments, and plays a key role in transcapillary fluid movement. A decreased COP increases transcapillary fluid movement, which leads to tissue edema and, combined with hemodilution, may compromise peripheral tissues oxygenation and end-organ perfusion^[3]^. The reduction of hemoglobin content has an impact during CPB on corrective action for red blood cell, hypothermia, and ultrafiltration uses, and it also has an impact on the oxygen delivery (DO₂) - there is ample evidence that an indexed DO₂< 280 ml/min/m^2^ exposes the patients to postoperative risk and incidence of acute kidney injury (AKI)^[4]^. The use of DN solution for the abovementioned aspects in the MiECC in particular for hemodilution variable appears in contrast, despite the literature being clearly in favor of this methodology in the context of conventional extracorporeal circulation. But there is another aspect we should ask ourselves when choosing myocardial protection solution:

1) Should it be chosen in relation to time according to the surgical technique?

2) Should it be chosen in relation to patient typology and multi-organ preservation during and after CPB?

From this point of view, there is no myocardial solution that integrates the answer to these two questions, and consequently the one that meets the benefits and objectives set by MiECC is the BC solution.

The literature on minimally invasive cardiac surgery, independently of the myocardial solution used, showed a significant association between cross-clamping time and mortality, low cardiac output syndrome, and AKI^[5]^, so moving towards a more physiological CPB such as the MiECC is perhaps the most reliable answer.
